# Berberine Protects C17.2 Neural Stem Cells From Oxidative Damage Followed by Inducing Neuronal Differentiation

**DOI:** 10.3389/fncel.2019.00395

**Published:** 2019-09-03

**Authors:** Jia-Wen Shou, Chun-Kai Cheung, Jian Gao, Wei-Wei Shi, Pang-Chui Shaw

**Affiliations:** ^1^School of Life Sciences, The Chinese University of Hong Kong, Shatin, Hong Kong; ^2^Li Dak Sum Yip Yio Chin R&D Centre for Chinese Medicine, The Chinese University of Hong Kong, Shatin, Hong Kong; ^3^Shenzhen Health Development Research Center, Shenzhen, China; ^4^State Key Laboratory of Research on Bioactivities and Clinical Applications of Medicinal Plants and Institute of Chinese Medicine, The Chinese University of Hong Kong, Shatin, Hong Kong

**Keywords:** berberine, neural stem cell, oxidative damage, differentiation, neuron

## Abstract

Neurodegeneration is the loss of structure and/or function of neurons. Oxidative stress has been suggested as one of the common etiology in most of the neurodegenerative diseases. Previous studies have demonstrated the beneficial effects of berberine in various neurodegenerative and neuropsychiatric disorders. In this study, we hypothesized that berberine could protect C17.2 neural stem cells (NSCs) from 2,2′-Azobis(2-amidinopropane) dihydrochloride (AAPH)-induced oxidative damage then promote neuronal differentiation. AAPH was used to induce oxidative damage. After the damage, berberine protected C17.2 cells were kept cultured for another week in differentiation medium with/without berberine. Changes in cell morphology were detected by microscopy and cell viability was determined by MTT assay. Real-time PCR and western blot analysis were performed to confirm the associated pathways. Berberine was able to protect C17.2 NSCs from the oxidative damage. It lowered the cellular reactive oxygen species (ROS) level in C17.2 cells via Nuclear Factor Erythroid 2-Related Factor 1/2 (NRF1/2) – NAD(P)H Quinone Dehydrogenase 1 (NQO-1) – Heme Oxygenase 1 (HO-1) pathway. It also down-regulated the apoptotic factors-Caspase 3 and Bcl2 Associated X (Bax) and upregulated the anti-apoptotic factor-Bcl2 to reduce cell apoptosis. Besides, berberine increased C17.2 cell viability via up-regulating Extracellular-signal-Related Kinase (ERK) and phosphor-Extracellular-signal-Related Kinase (pERK) expression. Then, berberine promoted C17.2 cell to differentiate into neurons and the differentiation mechanism involved the activation of WNT/β-catenin pathway as well as the upregulation of expression levels of pro-neural factors Achaete-Scute Complex-Like 1 (ASCL1), Neurogenin 1 (NeuroG1), Neuronal Differentiation 2 (NeuroD2) and Doublecortin (DCX). In conclusion, berberine protected C17.2 NSCs from oxidative damage then induced them to differentiate into neurons.

## Introduction

Neurodegeneration is the loss of structure and/or function of neurons, resulting in the death of neurons. Typical neurodegenerative diseases include Alzheimer’s disease (AD), Parkinson’s disease (PD), and Huntington’s disease (HD) (Gao and Hong, 2008). As the population ages, the economic burden from age-related health disorders also increases, necessitating measures to prevent or treat these disorders.

There are some common hallmarks among neurodegenerative diseases, such as synaptic dysfunction, oxidative stress, and inflammation (Chen X. et al., 2012). Emerging evidence suggests that oxidative stress may be one of main causes in neurodegeneration pathogenesis (Andersen, 2004; Kim et al., 2015). Oxidative stress can lead to impaired cellular functions and formation of toxic species, thus clearing reactive oxygen species (ROS) has been considered as an effective treatment measure toward neurodegenerative diseases (Van Raamsdonk et al., 2017). Besides, neuron loss is also a common feature of neurodegeneration (Zarow et al., 2003). There are a number of ways for neuronal demise, including apoptosis, necrosis, autophagic cell death and excitotoxicity (Gorman, 2008).

Neural stem cells (NSCs) can not only proliferate but also differentiate into neural cells such as neurons, astrocytes, and oligodendrocytes, and are therefore considered as multipotent (Kim, 2018). Some natural products like Tenuigenin (Chen Y. et al., 2012) and Incensole Acetate (El-Magd et al., 2018) have been reported to promote NSCs proliferation and increase differentiation *in vitro*. In addition, Asarone (Mao et al., 2015), Curcumin (Kim et al., 2008) and extract of *Ginkgo biloba* 761 (Tchantchou et al., 2007) showed the therapeutic effects toward AD mice via enhancing neural cell proliferation and neurogenesis. Thus promotion of neuronal proliferation and differentiation from NSCs should be taken into consideration when developing new anti-neurodegeneration medicines.

Berberine is an isoquinoline alkaloid, derived from the rhizome of *Coptis chinensis* (“Huang-Lian” in Chinese) of Family *Ranunculaceae*. It has been widely used for gastrointestinal infection (Feng et al., 2015). In recent years, berberine has been reported to be effective toward neurodegenerative diseases both *in vitro* and *in vivo* (Jiang et al., 2015). Berberine exerted neuroprotection effects toward SH-SY5Y, PC12 and N2a cells in different *in vitro* models of neurotoxicity including 6-hydroxydopamine, glutamate, hydrogen peroxide, oxygen-glucose deprivation, *N*-methyl-Daspartate-type glutamate receptor stimulation as well as CoCl_2_-induced hypoxia (Cui et al., 2009; Zhang et al., 2012; Bae et al., 2013; Sadeghnia et al., 2017). Besides, berberine also showed therapeutic effect toward AD via inhibition of monoamine oxidase B (MAO-B), cholinesterases and β-secretase activities, defense against damage from ROS, and reduction of the amyloid-beta genesis (Asai et al., 2007; Jung et al., 2009; Panahi et al., 2013). For PD, berberine could prevent neuron loss via decreasing cell apoptosis, inhibiting MAO-A and MAO-B activities and clearing ROS (Kong et al., 2001; Bae et al., 2013; Kim et al., 2014). Berberine can also inhibit neural cancer development via promoting neuronal differentiation of neuroblastoma cells (Naveen et al., 2016).

Neural cells exposed to ROS could result in severe cellular impairment, ultimately leading to neuronal death, which has been documented to play a significant role in cell loss during neurodegenerative disorders, such as stroke, PD, and AD (Loh et al., 2006). Azo compounds like AAPH are commonly used as free radical initiators (Niki, 1990). AAPH is a hydrophilic radical initiator and can continuously generate ROS. AAPH was reported to induce the oxidation of membrane lipids and proteins (Niki, 1990; Spiteller, 2006). Lipid peroxidation could lead to free radical-mediated injury that directly damages neural membranes and subsequently yields extensive cellular damage (Spiteller, 2006), which has been reported to be associated with the development of neurodegenerative disease like AD and PD (Shichiri, 2014).

Protecting neural cells from oxidative damage and promoting neurogenesis are two promising strategies for neurodegenerative patients. In this study, AAPH was used to induce oxidative damage. We aimed to elucidate whether berberine exerts both anti-oxidation and neurogenesis effects on AAPH-damaged C17.2 NSCs and find out the potential mechanisms.

## Materials and Methods

### Cell Culture and Reagents

Berberine (purity > 95%) was purchased from Cayman Chemical (Ann Arbor, MI, United States) and stored at −20°C. Vitamin C (or L-Ascorbic acid), Dulbecco’s Modified Eagle Medium (DMEM), fetal bovine serum (FBS), horse serum (HS), penicillin-streptomycin (PS) and trypsin were obtained from Thermo Fisher Scientific (Hong Kong). Dimethyl sulfoxide (DMSO), 3-(4,5-dimethylthiazol-2-yl)-2,5-diphenyltetrazolium bromide (MTT) and AAPH were purchased from Sigma-Aldrich (St. Louis, MO, United States). Antibodies for detecting the following proteins were used in this research: NRF-1 (Santa Cruz, sc-515360), NRF-2 (Santa Cruz, sc-365949), NQO-1 (Santa Cruz, sc-32793), HO-1 (Santa Cruz, sc-136960), Bax (Santa Cruz, sc-20067), Bcl2 (Santa Cruz, sc-23960), Caspase 3 (Cell Signaling, 9662S), Cleaved Caspase 3 (Cell Signaling, 9661S), ERK1/2 (Santa Cruz, sc-514302), pERK (Santa Cruz, sc-377400), Nestin (Santa Cruz, sc-23927), MAP2 (Santa Cruz, sc-74421), Olig 2 (Abcam, ab109186), GFAP (Santa Cruz, sc-33673), WNT3α (Santa Cruz, sc-74537), β-Catenin (Santa Cruz, sc-53483), β-Actin (Santa Cruz, sc-47778), TUBB3 (Santa Cruz, sc-80016), ASCL1 (Santa Cruz, sc-374104), NeuroG1 (Santa Cruz, sc-100332), NeuroD2 (Santa Cruz, sc-365896) and DCX (Santa Cruz, sc-271390).

C17.2 NSCs were obtained from Dr. David Walsh, Department of Anatomy, University of New South Wales, Australia. The proliferation medium and differentiation medium were prepared according to Li et al’s publication (Li et al., 2011) with some modifications. They are routinely cultured to 70–80% confluency in proliferation medium consisting of DMEM supplemented with 10% FBS, 5% HS, and 0.5% PS and cultured in standard humidified incubator (37°C and 5% CO_2_). The differentiation medium consisted of DMEM supplemented with 2.5% FBS, 1.25% HS, and 0.5% PS.

### Cytotoxic Effect of AAPH on C17.2 Cells

C17.2 cells (5 × 10^3^ per well) were seeded into 96-well plate with proliferation medium for 24 h then treated with AAPH at various concentrations (0.92, 1.85, 3.69, 7.38, 14.75, and 29.50 mM) for 12 h. An equivalent amount of DMSO (0.1%) was used as the vehicle control. Cell viability was detected via MTT assay. Briefly, MTT (5 mg/ml, 10 μl) was added to each well, and then cells were cultured for 4 h, followed by the removal of culture medium and the addition of 150 μl of DMSO. The absorbance was measured at 570 nm, and cell viability was calculated with the following equation: Cell viability (%) = Mean treatment OD/Control OD × 100%. IC 50 was calculated from GraphPad Prism Version 6.0C (GraphPad Software, La Jolla, CA, United States).

### Protective Effect of Berberine Toward C17.2 Cells

C17.2 cells (5 × 10^3^ per well) were seeded into 96-well plate with proliferation medium for 24 h. AAPH at the concentration of 7.38 mM could induce about 40% cell death, thus this concentration of AAPH was selected for the following experiments. Berberine with various concentrations (0.85, 1.69, 3.38, 6.75, 13.5, 27.0, 54.0 μM) as well as AAPH (7.38 mM) were added to C17.2 cells for 12 h. Cell viability was detected by MTT assay.

### Neuronal Proliferation and Differentiation of C17.2 Cells

C17.2 cells (2 × 10^3^ cells per well) were seeded into 6-well plate with proliferation medium (DMEM supplemented with 10% FBS, 5% HS, and 0.5% PS) for 24 h. The cells were then incubated with berberine (0.85, 1.69, 3.38, 6.75, 13.5 μM) in differentiation medium (DMEM supplemented with 2.5% FBS, 1.25% HS, and 0.5% PS) for 6 days. An equivalent amount of DMSO (0.1%) was used as the vehicle control. Differentiation of C17.2 NSCs was morphologically determined by the previously described criteria with minor modifications (Li et al., 2011). Briefly, the morphologically differentiated cells were counted from randomly selected non-overlapping microscopic fields under a phase-contrast microscope (Olympus). The percentages of morphologically differentiated cells in the berberine treatment groups were compared against the DMSO (0.1%) vehicle control. Cell having a neurite extension of length at least twice the diameter of its cell body was considered as differentiated. Cell differentiation percentage (%) = (differentiated cells number in one randomly selected non-overlapping microscopic fields/average total cell number in this filed) × 100%. In total nine randomly selected non-overlapping microscopic fields will be counted for calculation.

**FIGURE 1:**
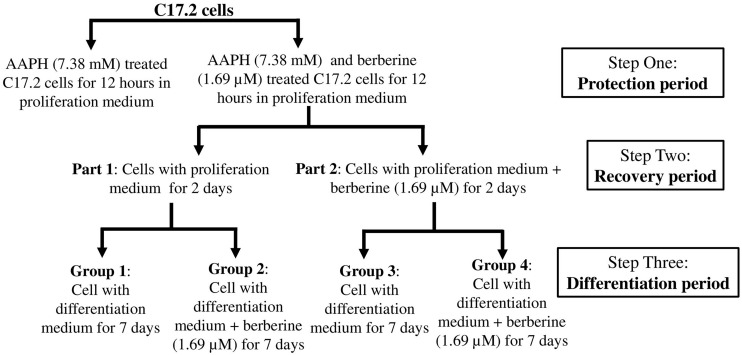
Work flow showing the design of experiments.

Then AAPH was used to generate the oxidation damage and berberine was added to protect the cells and also induce neuronal differentiation. The workflow of this part was shown in Figure 1.

Step one was the protection period. C17.2 cells (2 × 10^3^ cells per well) were seeded into eight 25-cm^3^ flask with proliferation medium for 24 h. 1.69 μM berberine not only showed the second best protective effects from AAPH damage but also showed the second best effect to promote cell differentiation. Thus we chose 1.69 μM berberine for the following experiments. Berberine (1.69 μM) and AAPH (7.38 mM) were added to treat C17.2 cells for 12 h. Cells treated only with AAPH were set as control.

Step two was the recovery period. Berberine and AAPH treated cells were kept cultured for the following experiments. Cells were washed with cold PBS twice then divided into two parts (part 1 and part 2, four flasks in each part). One part of cells was treated with berberine (1.69 μM) in proliferation medium for 2 days, and the other one was treated with equivalent amount of DMSO in proliferation medium for 2 days.

Step three was the differentiation period. Each part was sub-divided into two groups, and in total there were four groups. Group 2 and group 4 were treated with berberine (1.69 μM) in differentiation medium for 7 days; group 1 and group 3 were treated with equivalent amount of DMSO in differentiation medium.

Images of cells were taken under a phase-contrast microscope (Olympus). Total RNA and total protein were extracted from all these three steps and stored at −80°C for subsequent analysis.

### Flow Cytometry Analysis

C17.2 cells (1 × 10^6^/well) were cultured in 6-well plate in proliferation medium for 24 h. AAPH (7.38 mM) and berberine (1.69 mM) was added to treat C17.2 cell for 12 h, while only AAPH (7.38) added was set as control. After 12-h treatment, cells were collected and stained with FITC Annexin V and propidium iodide (PI) in the dark using a FITC Annexin V and PI Apoptosis Kit (A026, GeneCopoeia), according to the manufacturers’ instruction. After being washed, the apoptotic cells were determined by a BD FACSCanto^TM^ II Flow Cytometry System.

### ROS Detection

The ROS levels were detected in step one and two. The intracellular formation of ROS was detected using the fluorescent probe H_2_DCFDA, according to a published method (Wu and Yotnda, 2011). Briefly, after treatment, the cells were washed with PBS twice, loaded with 10 μM H_2_DCFDA, and incubated for 30 min at 37°C in dark. Relative fluorescence intensity was monitored by using a CLARIOstar Microplate Reader (BMG LABTECH) with 485 nm excitation and 520 nm emission.

### Real-Time PCR Analysis

Expression of target genes was analyzed by quantitative PCR. The primers were synthesized by Tech Dragon Limited (Table 1). Total RNA was purified using TRIzol reagent (Invitrogen) and quantified using QIAxpert (QIAGEN GmbH). RNA extracted from step one was used for detecting anti-oxidant genes (*NRF-1*, *NRF-2*, *HO-1*, and *NQO-1*) and apoptotic genes (*Bax*, *Bcl2*, *Caspase 3*) mRNA levels. RNA extracted from step two was used for detecting pro-neural markers (*ASCL1*, *NeuroG1*, *NeuroD2*, and *DCX*) mRNA levels. RNA extracted from step three was used for detecting neuronal markers (*Nestin*, *MAP2*, *TUBB3*, *GFAP*, and *Olig2*) and neuronal differentiation related genes (*WNT3α* and *β-Catenin*) mRNA levels. After reverse transcription, real-time PCR reactions were performed using an ABI Step One Plus (Applied Biosystems, United States) with Luna Universal SYBR real-time PCR Master Mix (NEB, United States). The reaction system used for the real-time PCR mixture was prepared according to the manufacturer instruction. The real-time PCR protocol was set as follow: initial denaturation at 95°C for 1 min, then 40 cycles of denaturation at 95°C for 15 s and annealing at 60°C for 30 s. The melt curve protocol followed with 15 s at 95°C and then 10 s each at 0.2°C increments between 60°C and 95°C. The mRNA expression level of *GAPDH* was used as the internal standard. The relative expression level was calculated by comparison of the tested groups with control group using the 2^–ΔΔ*CT*^ method.

**TABLE 1:** Real time PCR primers.

	**Primer sequence (5′-3′)**	**Accession number**
*Nestin*	F: ACCTATGTCTGAGGCTCCCTATCCTA	NM_016701.3
	R: GAGGTTGGATCATCAGGGAAGTG	
*MAP2*	F: CTCTGCCTCTAGCAGCCGAA	NM_001310634.1
	R: CACCACTTGCTGCTTCCTCC	
*Olig2*	F: GGCGGTGGCTTCAAGTCATC	NM_016967.2
	R: TAGTTTCGCGCCAGCAGCAG	
*TUBB3*	F: CTCAGTCCTAGATGTCGTGC	NM_023279.3
	R: GCGGAAGCAGATGTCGTAGA	
*GFAP*	F: AGAAAACCGCATCACCATTC	NM_010277.3
	R: TCACATCACCACGTCCTTGT	
*Wnt3α*	F: AGTGCCAGCACCAGTTCC	NM_009522.2
	R: CATGGACAAAGGCTGACTCC	
β*-Catenin*	F: TGGACCCTATGATGGAGCATG	NM_001165902.1
	R: GGTCAGTATCAAACCAGGCCAG	
*NRF-1*	F: GCACCTTTGGAGAATGTGGT	NM_010938.4
	R: CTGAGCCTGGGTCATTTTGT	
*NRF-2*	F: CCATTTACGGAGACCCACCGCCTG	NM_010902.4
	R: CTCGTGTGAGATGAGCCTCTAAGCGG	
*HO-1*	F: AAGCCGAGAATGCTGAGTTCA	NM_010442.2
	R: GCCGTGTAGATATGGTACAAGGA	
*NQO-1*	F: GGACATGAACGTCATTCTCT	NM_008706.5
	R: TTCTTCTTCTGCTCCTCTTG	
*Bax*	F: ACAGATCATGAAGACAGGGG	NM_007527.3
	R: AAAGTAGAAGAGGGCAACCA	
*Bcl-2*	F: GAGAGCGTCAACAGGGAGATG	NM_009741.5
	R: CCAGCCTCCGTTATCCTGGA	
*Caspase 3*	F: TGACTGGAAAGCCGAAACTC	NM_009810.3
	R: AGCCTCCACCGGTATCTTCT	
*ASCL1*	F: ACTTGAACTCTATGGCGGGTT	NM_008553.5
	R: CCAGTTGGTAAAGTCCAGCAG	
*NeuroG1*	F: CCAGCGACACTGAGTCCTG	NM_010896.2
	R: CGGGCCATAGGTGAAGTCTT	
*NeuroD2*	F: CAAGAAGCGCGGGCCGAAGA	NM_010895.3
	R: TTGGCCTTCTGTCGCCGCAG	
*DCX*	F: TTCGTAGTTTTGATGCGTTGCT	NM_001110224.1
	R: GAGGCAGGTTAATGTTGTCAG	
*GAPDH*	F: GCACAGTCAAGGCCGAGAAT	NM_001289726.1
	R: GCCTTCTCCATGGTGGTGAA	

### Western Blot Analysis

The protein expression levels of NRF-1, NRF-2, NQO-1, HO-1, Bax, Bcl2, Caspase 3, Cleaved Caspase 3, ERK1/2, pERK, Nestin, MAP2, TUBB3, Olig2, WNT3α, β-Catenin, ASCL1, NeuroG1, NeuroD2, DCX and β-Actin in C17.2 cells were determined by western blot analysis. Proteins extracted from step one will be used for detecting NRF-1, NRF-2, NQO-1, HO-1, Bax, Bcl2, Caspase 3 and Cleaved Caspase 3 expression levels. Proteins extracted from step two will be used for detecting ERK1/2, pERK, ASCL1, NeuroG1, NeuroD2 and DCX expression levels. Proteins extracted from step three will be used for detecting Nestin, MAP2, TUBB3, Olig2, WNT3α and β-Catenin expression levels. Total protein (15 or 30 μg) were fractionated by electrophoresis on 8 or 10% polyacrylamide gels and transferred to PVDF membrane. Then membranes were blocked in 5% BSA at 4°C overnight, the membranes were incubated with primary antibodies at 4°C for 8 h, subsequently incubated with secondary antibodies for 1 h at room temperature after thoroughly washing. Target proteins were detected using an ECL kit (GE Healthcare) and visualized by a ChemiDoc Imaging System (Biorad), and signals were quantified using ImageJ (version 1.51m9) analysis.

### Immunofluorescence Staining

Cells of group 1, group 2, group 3, and group 4 were cultured in confocal dishes as above. Then cells were fixed with 4% paraformaldehyde for 30 min at room temperature, washed with phosphate-buffered saline (PBS, pH7.6) and permeabilized with 0.1% Triton X-100 for 10 min at room temperature. Subsequently cells were blocked with 2% BSA solution for 60 min at room temperature. Cells were incubated with primary antibodies at 4°C overnight. The primary antibodies were anti- Nestin (Santa Cruz, 1:100), anti-MAP2 (Santa Cruz, 1:100), anti-GFAP (Santa Cruz, 1:100) and anti-Olig 2 (Abcam, 1:100). The cells were washed three times with PBS and incubated with Alexa Fluor 488 secondary antibody at room temperature for 1 h. Cells were counterstained with 0.05 ng/ml 4′,6-diamidino-2-phenylindole (DAPI, Sigma) for 10 min to visualize nucleus. Finally, all images were captured with a Leica TCS SP8 Confocal Microscope (Leica).

### Statistical Analysis

The statistical analyses were conducted using one-way ANOVA and Student’s *t*-test with the GraphPad Prism Version 6.0C (GraphPad Software, La Jolla, CA, United States). The data were expressed as means ± SEM. *P* values less than 0.05 were considered statistically significant.

## Results

### Berberine Protected C17.2 Cells From AAPH Damage

We initially treated C17.2 cells with different concentrations of berberine (0.85, 1.69, 3.38, 6.75, 13.5, 27.0, 54.0 μM) for 12 and 24 h. The results showed cell viability was not increased after berberine treatment for 12 h (Supplementary Figure S1), however, after 24-h treatment, 0.85, 1.69, and 3.38 μM berberine showed higher cell viability when compared that of control (Supplementary Figure S2). If we selected 24-h treatment subsequently, it would be difficult to differentiate the anti-AAPH effect from the cell viability promoting effect of berberine. Thus we treated C17.2 cell with berberine and/or AAPH for 12 h in the following experiments.

**FIGURE 2:**
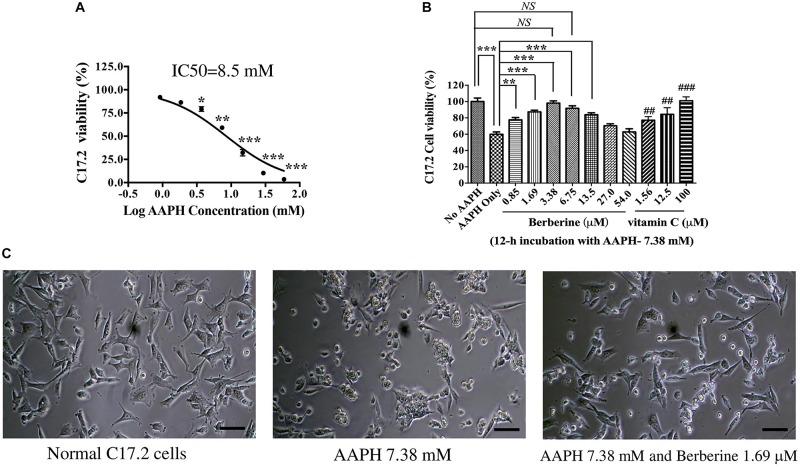
The protective effect of berberine toward AAPH-damaged C17.2 neural stem cells. **(A)** AAPH induced C17.2 cell death. **(B)** Berberine protected C17.2 cell from AAPH (7.38 mM) induced oxidative damage. 1.25, 12.5, and 100 μM of vitamin C was used as the positive control. **(C)** Cell morphology after AAPH (7.38 mM) and berberine (1.69 μM) treatment. The data represent the mean ± SEM. ## and ### indicated to compare with AAPH only. ^∗^*P* < 0.05, ^∗∗^ or ## *P* < 0.01 and ^∗∗∗^ or ###*P* < 0.001. NS, no significance.

AAPH was used to induce oxidative damage. After C17.2 cells were treated with various concentrations of AAPH for 12 h, cell viability was detected by MTT assay. AAPH induced C17.2 death following a dose-dependent manner (Figure 2A). The IC50 of AAPH toward C17.2 was 8.50 mM.

AAPH at a concentration of 7.38 mM was selected in subsequent studies, under which there showed about 60% cell viability. AAPH (7.38 mM) and berberine with different concentrations (0.85, 1.69, 3.38, 6.75, 13.5, 27.0, 54.0 μM) were incubated with C17.2 cells for 12 h, while 1.25, 12.5, and 100.0 μM Vitamin C was used as the positive control. Vitamin C, a potent antioxidant, showed the dose-dependent manner to protect C17.2 cells from AAPH-induced damage (Figure 2B). Similarly, berberine protected cells from oxidative damage with a dose-dependent manner. The cell viability of C17.2 cells treated with vehicle was set as 100%. AAPH (7.38 mM) treated cells showed the viability of 60.4 ± 2.6%, while berberine at 3.38 μM showed the strongest protective effect, with 94.9 ± 3.27% cells viable (Figure 2B), followed by 1.69, 6.75, and 0.85 μM berberine. Interestingly, higher concentration of berberine at 27.0 and 54.0 μM did not show protective effect (Figure 2B).

Cells after AAPH treatment showed obvious morphological changes, becoming rounded, shrunken, and more loosely attached to the cell culture dish surface (Figure 2C), while in the presence of berberine, many cells appeared normal in shape (Figure 2C).

### Berberine Induced Normal C17.2 Cells Differentiation

**FIGURE 3:**
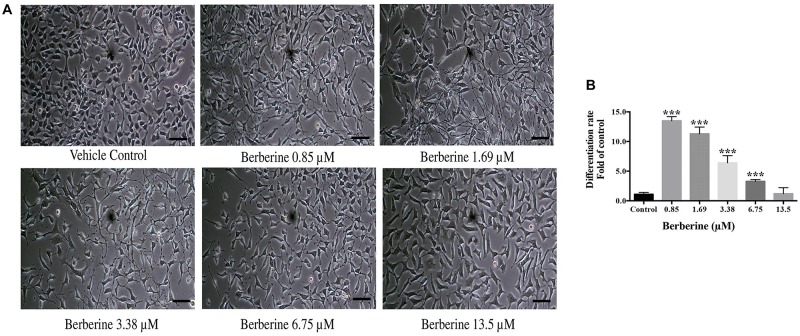
Berberine induced the differentiation of C17.2 neural stem cells. **(A)** Images of differentiated C17.2 cells. **(B)** Differentiation rate after induction by berberine. The data represent the mean ± SEM. ^∗∗∗^*P* < 0.001. Scale bar represents 10 μm.

Berberine at protective concentrations of 0.85, 1.69, 3.38, 6.75, and 13.5 μM were used to induce C17.2 cell differentiation. Cells treated with berberine displayed differentiated morphologies of condensed cell bodies and extended neurite outgrowths, in contrast to the flattened and irregular cell bodies of the undifferentiated cells observed in the vehicle control (Figure 3A).

The morphologically differentiated cells were counted from nine randomly selected non-overlapping microscopic fields and the percentage of differentiation was counted versus vehicle control. Among the selected concentrations, berberine promoted more neural differentiations at lower concentration (Figure 3B). Compared with the control group, 0.85 and 1.69 μM berberine showed 13.5 ± 1.3 and 11.3 ± 2.0 folds more differentiated cells, respectively, when compared with that of control.

### Berberine Protected C17.2 Cells From AAPH Damage Then Induced Neuronal Differentiation

**FIGURE 4:**
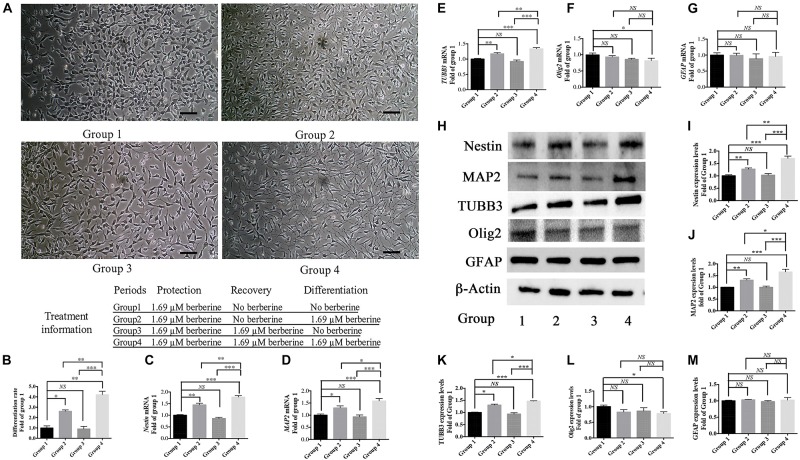
Berberine induced neuronal differentiation of AAPH-damaged C17.2 cells. **(A)** Cell differentiation in group 1, group 2, group 3, and group 4. **(B)** Differentiation rate in group 1, group 2, group 3, and group 4. **(C–G)** mRNA levels of *Nestin* **(C)**, *MAP2* **(D)**, *TUBB3* **(E)**, *Olig2* **(F)**, and *GFAP* **(G)** in Group 1, 2, 3, and 4. **(H)** Western blot analysis of Nestin, MAP2, TUBB3, Olig2, and GFAP in Group 1, 2, 3, and 4. **(I–M)** Quantification of protein blots. Group 1 was set as the control. The data represent the mean ± SEM. ^∗^*P* < 0.05, ^∗∗^*P* < 0.01, and ^∗∗∗^*P* < 0.001.

From the above results, 1.69 μM berberine not only protected C17.2 cells from AAPH damage but also showed better effect to promote cell differentiation. Thus, berberine (1.69 μM) and AAPH (7.38 mM) were added to treat C17.2 cells for 12 h, and cells treated only with AAPH were set as control. In Step One, C17.2 cells were treated with berberine (1.69 μM) and AAPH (7.38 mM), cells treated only with AAPH (7.38 mM) was set as control. Then berberine -AAPH-treated cells were kept cultured to induce differentiation. In Figure 4A, cells in group 2 and group 4 showed significant neural differentiation. Differentiation rate was shown in Figure 4B with group 1 set as the control. Cells in group 4 showed the highest differentiation rate (4.2 ± 0.3 folds), then followed by group 2 (2.6 ± 0.1 folds). Cells in both group 1 and group 3 did not show obvious cell differentiation.

**FIGURE 5:**
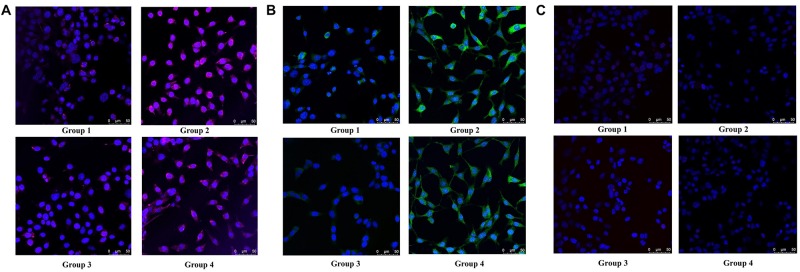
Immunofluorescence staining of Nestin, MAP2 and Olig2 in group 1, group 2, group 3, and group 4. **(A)** Nestin (magenta) and DAPI (blue) fluorescent staining. **(B)** MAP2 (green) and DAPI (blue) fluorescent staining. **(C)** Olig2 (red) and DAPI (blue) fluorescent staining.

To determine the type of neural cells resulted from berberine induction, we extracted total RNA and protein from step 3 (group 1, 2, 3, and 4) and detected the expression levels of Nestin (a NSC marker), MAP2 (a neuron marker), TUBB3 (a neuron marker), Olig2 (an oligodendrocyte marker) and GFAP (an astrocyte marker) via real-time PCR and western blot analysis. Real-time PCR showed berberine could upregulate the mRNA levels of *Nestin*, *MAP2*, and *TUBB3*, while *GFAP* mRNA level remained unchanged; berberine seemed to slightly decrease the mRNA level of *Olig2* (Figures 4C–G). In addition, all these changes in group 4 were significantly higher than those in group 2. Western blot analysis confirmed that berberine could increase the expression levels of Nestin, MAP2 and TUBB3 but did not alter the GFAP expression level; same as real-time PCR results, from group 1 to 4 berberine slightly reduced Olig2 expression with group 1 showing the highest expression (Figures 4H–M). For better confirmation of neural cell types, we also performed immunofluorescence staining for neural makers. Results showed that immunofluorescence staining (Nestin, MAP2, GFAP, and Olig2) shared the similar results with western blot analysis. As shown in Figures 5A,B, there showed the most Nestin positive (magenta) or MAP2 positive (green) cells in group 4, then followed by group 2, while fewer positive cells were detected in group 1 and group 3. As for GFAP, few positive cells were detected in these four groups (data not shown). Obvious Olig2 positive (red) cells were detected in group 1 (Figure 5C), whereas almost no Olig2 positive cell was found in the other three groups. These findings indicated berberine could protect C17.2 cells from AAPH damage then induce the differentiation to neurons rather than to oligodendrocytes or astrocytes.

**FIGURE 6:**
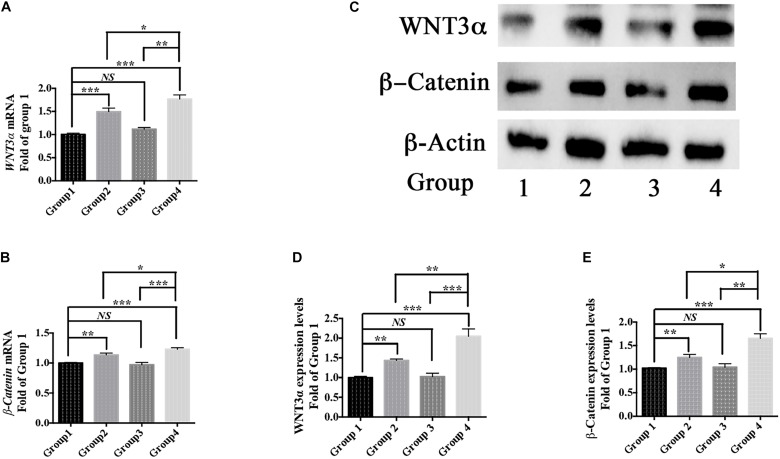
Differentiation pathway. **(A,B)** mRNA levels of *WNT3α* **(A)** and *β-Catenin* **(B)** in Group 1, 2, 3, and 4. **(C)** Western blot analysis of WNT3a and b-Catenin in Group 1, 2, 3, and 4. **(D,E)** Quantification of protein blots. Group 1 was set as the control. The data represent the mean ± SEM. ^∗^*P* < 0.05, ^∗∗^*P* < 0.01, and ^∗∗∗^*P* < 0.001.

Moreover, berberine increased the expression of WNT3α and β-Catenin at both transcriptional and translational levels (Figures 6A–E). Hence, berberine could promote C17.2 cell differentiation via, at least in part, WNT/β-Catenin pathway.

### Berberine Showed the Protective Effect via Anti-oxidant and Anti-apoptotic Pathways

**FIGURE 7:**
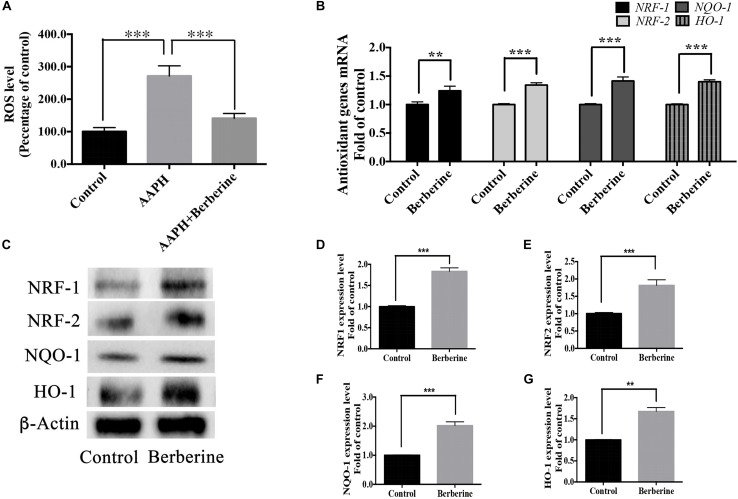
Antioxidant pathway of berberine. **(A)** Detection of ROS levels in Step one. **(B)** mRNA levels of antioxidant genes *NRF-1, NRF-2, HO-1*, and *NQO-1*. **(C)** Western blot analysis of NRF-1, NRF-2, HO-1, and NQO-1 in Step one. **(D–G)** Quantification of protein blots. Group 1 was set as the control. The data represent the mean ± SEM. ^∗∗^*P* < 0.01 and ^∗∗∗^*P* < 0.001.

ROS levels were detected in step one (protection period). As shown in Figure 7A, the addition of AAPH (7.38 mM) significantly increased the ROS level to 285.5 ± 23.9%, whereas berberine (1.69 μM) was able to decrease this elevated ROS level. Real-time PCR analysis of RNA collected from step one showed that berberine could increase the anti-oxidant genes (*NRF-1*, *NRF-2*, *HO-1*, and *NQO-1*) expression levels by 24.2 ± 3.6%, 34.3 ± 3.8%, 41.4 ± 7.1%, and 40.2 ± 3.3%, respectively (Figure 7B). Western blot analysis confirmed the effect of berberine to upregulate these antioxidant proteins (Figures 7C–G).

**FIGURE 8:**
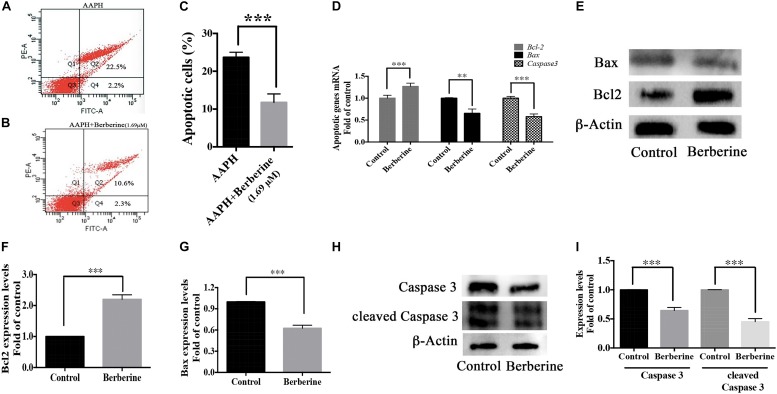
Antiapoptotic pathway of berberine. **(A–C)** Cell apoptosis detected by flow cytometry, **(A)** AAPH (7.38 mM) treated cells; **(B)** AAPH (7.38 mM) and berberine (1.69 μM) treated cells. **(C)** Apoptotic cells percentage (Q2 + Q4, calculated from three independent experiments). **(D)** mRNA levels of anti-apoptotic gene (*Bcl-2*) and apoptotic genes (*Bax* and *Caspase 3*) in Step one. **(E)** Western blot analysis of Bcl-2 and Bax in Step one. **(F,G)** Quantification of protein blots. **(H)** Western blot analysis of Caspase 3 and cleaved Caspase 3 in Step one. **(I)** Quantification of protein blots. The data represent the mean ± SEM. ^∗^*P* < 0.05, ^∗∗^*P* < 0.01, and ^∗∗∗^*P* < 0.001.

Berberine also decreased the ROS-induced apoptosis in AAPH-damaged C17.2 cells. With flow cytometry, 12-h AAPH treatment could lead to 23.4 ± 0.7% apoptotic cells, while in the presence of berberine (1.69 μM), the apoptotic cells were reduced to 10.9 ± 2.2% (Figures 8A–C). Besides, berberine upregulated anti-apoptotic gene (*Bcl-2*) expression and downregulated apoptotic genes (*Bax* and *Caspase 3*) expressions at transcriptional and translational levels (Figures 8D–I) in step one.

### Berberine Promoted Cell Viability During Recovery Period

**FIGURE 9:**
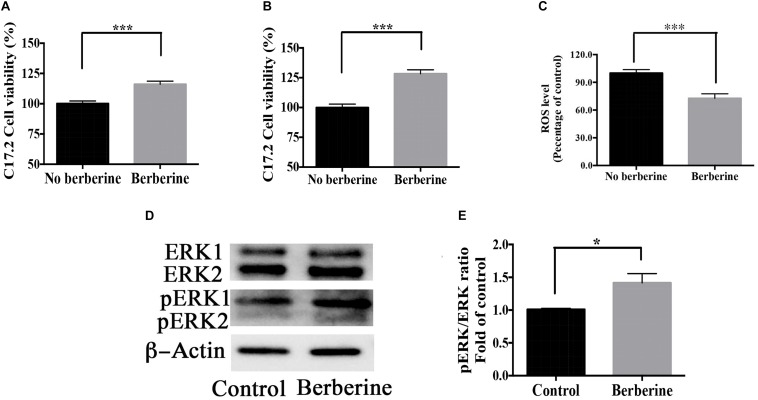
Berberine promoted cell viability during recovery period. **(A,B)** C17.2 cell viability after berberine treatment on recovery day 1 **(A)** and day 2 **(B)** in Step Two. **(C)** ROS level detection on recovery day 2 in Step Two. **(D)** Western blot analysis of ERK and pERK in Step Two. **(E)** Quantification of protein blots. The data represent the mean ± SEM. ^∗^*P* < 0.05, ^∗∗^*P* < 0.01, and ^∗∗∗^*P* < 0.001.

Cell viability during recovery period was detected by MTT assay. On recovery day 1, berberine increased the cell viability by 16.1 ± 2.6% (Figure 9A); on day 2, by 30.5 ± 4.1% (Figure 9B). Berberine reduced the ROS level by 28.7 ± 2.4% on day 2 (Figure 9C), while increased both ERK and pERK expression during the recovery period (Figures 9D–F).

### Berberine Promoted the Expression of Pro-neural Markers During the Recovery Period

**FIGURE 10:**
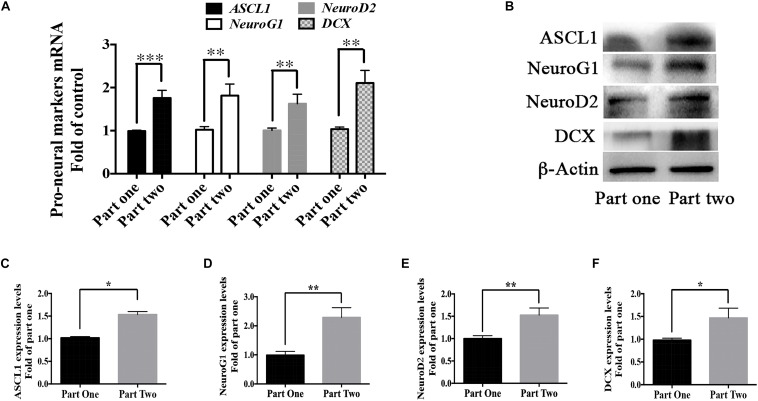
Berberine promoted the expression of pro-neural markers during the recovery period. **(A)** mRNA levels of pro-neural markers (*ASCL1*, *NeuroG1*, *NeuroD2*, and *DCX*) were detected in Step Two via real-time PCR analysis. **(B)** Western blot analysis of ASCL1, NeuroG1, NeuroD2, and DCX. **(C–F)** Quantification of protein blots. The data represent the mean ± SEM. ^∗^*P* < 0.05, ^∗∗^*P* < 0.01, and ^∗∗∗^*P* < 0.001.

As shown in Figure 1, cells in part 1 (step two) were evenly divided into two groups in step 3- Group 1 and 2; cells in part 2 (step two) were also evenly divided into two groups in step 3- Group 3 and 4. The difference between Group 2 and Group 4 was whether berberine was added in the recovery period (step two). But there showed higher differentiation rate in Group 4 than that in Group 2. We suspected the treatment of berberine in step two might affect the neuronal differentiation in step three. Thus, mRNA levels of some pro-neural markers were detected from the RNA extracted from step two (part 1 and part 2). There showed higher levels of pro-neural markers in part two than those in part one. In part two there increased the mRNA level of pro-neural markers *ASCL1*, *NeuroG1*, *NeuroD2*, and *DCX* by 75.9 ± 17.7%, 81.4 ± 11.5%, 62.5 ± 12.1%, and 106.1 ± 15.9% respectively (Figure 10A), when compared with part one. Moreover, the expression upregulation of these pro-neural markers had also been confirmed via western blot analysis (Figures 10B–F).

## Discussion

For treating neurodegenerative diseases, it is helpful to re-generate normal cells that are lost in patients and facilitate the production of neurons or glia from endogenous NSCs. Thus, molecules that promote endogenous NSC proliferation and neurogenesis are useful for treating neurodegenerative diseases.

Natural products are a rich source of compounds for drug discovery and Artemisinin for treating malaria and taxol for cancer are famous examples (Klayman, 1985; Holmes et al., 1991). Phytochemicals have recently been recognized as alternatives for treating neurodegenerative diseases such as AD and PD (Choi and Choi, 2015; Shal et al., 2018). For examples, Casticin (Liou et al., 2018), Ginsenoside Rg1 (Chen et al., 2018), and Bryostatin-1 (Safaeinejad et al., 2018) have been known to show anti-oxidative and anti-inflammatory effects and to increase cell survival and promote neurogenesis. Our data exhibited that berberine might be another promising drug candidate for treating neurodegeneration.

In this study, we treated C17.2 NSCs with AAPH to establish an *in vitro* neurodegeneration model. As shown in Figure 1, we performed this research for the protection, recovery and differentiation periods. In the protection period, berberine exerted anti-oxidation effect toward C17.2 cells exposed to AAPH. We set vitamin C as positive control and found that 3.38 μM berberine showed the comparative anti-AAPH effect with that of 100 mM vitamin C. After AAPH and berberine treatment, we recorded the cell morphology change. Normal C17.2 cells showed a flattened, elongated, fibroblast-like morphology, while AAPH-treated C17.2 cells exhibited round and shrunken shape, and more loosely attached to the cell culture dish surface, which was in accordance with C17.2 cell shape after oxidative damage (like AAPH, paraquat, 2,3-Dimethoxy-1,4-naphthoquinone) as recorded (Xia et al., 2005; Mutsaers and Tofighi, 2012; Alural et al., 2015). However, the presence of berberine kept most of the C17.2 cells unchanged in shape when exposed to AAPH. Thus, these two aspects offered evidence for showing the anti-oxidative effect of berberine toward AAPH-treated C17.2 cells.

Besides the morphological change, the excessive ROS resulted from AAPH could cell apoptotic death (Li et al., 2014). As shown in our results, berberine could reduce the AAPH-upregulated ROS levels and decrease apoptotic cell percentage, which showed another aspect of the anti-AAPH of berberine.

Berberine could also elevate the expression of the antioxidant genes like *NRF1*, *NRF2*, *NQO1*, *HO-1*. *NRF1* and *NRF2* (also known as Nfe2l1 and Nfe2l2) are master regulators for genes induced by oxidative stress (Ohtsuji et al., 2008). Both *NRF1* and *NRF2* are responsible for cellular redox balance and protective antioxidant responses. They have been shown to regulate enzymes involved in detoxification such as NQO1) and HO-1 (Ohtsuji et al., 2008; Sahoo et al., 2016). HO-1 expression is inducible in response to various forms of cellular damage and it can exhibit substantial anti-oxidative and anti-apoptotic effects (Kirkby and Adin, 2006), thus HO-1 induction has been considered as one of cellular defensive mechanisms. NQO1, the superoxide scavenger, is a flavoprotein that catalyzes the reduction of quinones, quinone amines and azo dyes, thereby protecting cells from oxidative stress triggered by these compounds (Xie et al., 1995; Siegel et al., 2004).

ROS also plays an important role in apoptosis induction (Redza-Dutordoir and Averill-Bates, 2016). The apoptotic cell percentage was detected using FITC Annexin V Apoptosis Detection Kit. AAPH could result in C17.2 cell apoptosis while berberine could reduce the apoptosis significantly. Elevated level of ROS causes oxidation of the mitochondrial pores thereby disrupting the mitochondrial membrane potential, resulting in the release of cytochrome C and the activation of caspases (Engel and Evens, 2006). In addition, ROS-induced apoptosis is also involved with the increased Bax expression and the decreased Bcl2 expression (Kaushik et al., 2015; Liu et al., 2016). We found that berberine not only inhibited the activation of Caspase 3 but also downregulated Bax expression and upregulated Bcl2 expression to protect cells from apoptosis. Thus, berberine might protect the AAPH-treated cells via anti-oxidation and anti-apoptosis pathways.

Then berberine and AAPH treated cells were kept cultured for 2-day recovery with/without berberine. According to our observation (data not shown), more dead cells and less differentiated cells were found without this recovery period. The effect of berberine added during this recovery period included: (1) to reduce the ROS level and (2) to promote cell viability. Berberine increased cell viability via upregulating ERK and pERK expression, which plays a critical role in the regulation of cell proliferation (Lu and Xu, 2006).

Owing to significant loss of neurons in neurodegenerative patients, neurogenesis might be a promising way to develop new anti-neurodegeneration medicines. In the differentiation period, when compared group 1 with group 2 (or group 3 with group 4), we found that berberine could promote AAPH-damaged C17.2 cells to show distinctly different morphological phenotypes with visual outgrowth of neurites. The differentiated cell morphology was similar to differentiated C17.2 cells in other publications (Li et al., 2011; Lundqvist et al., 2013). For determining the cell types after differentiation, real-time PCR, western blot analysis and immunofluorescence staining were used to detect the concerned neural cell markers. Nestin is an intermediate filament protein that is known as a neural stem/progenitor cell marker (Suzuki et al., 2010). MAP2 and TUBB3 are two biomarkers for neurons and they were selected as the biomarker for neuronal differentiation (Attoff et al., 2016; Marei et al., 2018). GFAP is the most commonly used indicator for astrocytes differentiated from NSCs (Attoff et al., 2016). Olig2 is up-regulated in oligodendrocytes and often used as a marker for oligodendrocytes (Koutsoudaki et al., 2016). When compared group 1 with group 2 (or group 3 with group 4), the addition of berberine upregulated Nestin expression, suggesting berberine also promoted C17.2 cells proliferation. Berberine also increased neuron markers MAP2 and TUBB3 expressions, which indicated that berberine promoted C17.2 cells to differentiate into neurons. Others have also shown the increase of MAP2 and TUBB3 expression levels when C17.2 cells were differentiated into neurons (Li et al., 2011; Lundqvist et al., 2013). GFAP expression was not altered by berberine, meaning berberine did not promote C17.2 cells to turn into astrocytes. While for Olig2 expression, there showed a reduction trend after berberine treatment (compare group 1 with group 2 or compare group 3 with group 4). From the recent publication, Olig2 expression level started to decrease over time after initiating neuronal differentiation (Xu et al., 2019). In this study, we found that the Olig2 expressions showed a reduction trend after berberine treatment (compare group 1 with group 2 or compare group 3 with group 4). The decrease of Olig2 expression is also an evidence supporting that berberine could initiate the neuronal differentiation of C17.2 cells. We therefore concluded that berberine promoted AAPH-damaged C17.2 NSCs to differentiate into neurons rather than astrocytes or oligodendrocytes. In addition, we found that the differentiation mechanism involved the WNT/β-Catenin pathway. WNT/β-Catenin pathway regulates stem cell pluripotency and cell fate decision during development (Clevers and Nusse, 2012). This pathway plays an important role in neuronal differentiation (Hirabayashi et al., 2004; Kondo et al., 2011).

We also found that there were more differentiated cells in group 4 than in group 2. The only difference between group 2 and group 4 was the addition of berberine during the recovery period (seen in Figure 1). Thus berberine added during the recovery period could promote neural differentiation. We proposed that berberine might regulate pro-neural markers in the recovery period. Pro-neural markers have been shown to initiate the development of neuronal lineages and to promote the generation of progenitors that are committed to differentiation (Bertrand et al., 2002). Determination of neuron fates from NSCs during brain development depends on some basic helix-loop-helix (bHLH) factors like ASCL1, NeuroG1 and NeuroD2 (Morrison, 2001; Ross et al., 2003; Henke et al., 2009). In addition, DCX, a protein facilitating microtubule polymerization, is expressed in migrating neuroblasts and immature neurons and has also been considered as a marker for early immature neurons (Henke et al., 2009). From our results berberine increased the expression levels of pro-neural markers like ASCL1, NeuroG1, NeuroD2, and DCX to promote neuronal differentiation.

In summary, the present study demonstrated that berberine protected C17.2 NSCs from AAPH induced damaged and then promoted them to differentiate into neurons, suggesting that berberine is a promising chemical for treating neurodegeneration.

## Data Availability

The datasets generated for this study are available on request to the corresponding author.

## Author Contributions

P-CS designed the experimental plan. J-WS performed the experiments, collected the data, and drafted the manuscript. JG performed the flow cytometry experiments and analyzed the data. J-WS, C-KC, and W-WS analyzed and interpreted the data. All authors approved the final version of the manuscript.

## Conflict of Interest Statement

The authors declare that the research was conducted in the absence of any commercial or financial relationships that could be construed as a potential conflict of interest.
