# Supplementary material for: Berberine Protects C17.2 Neural Stem Cells From Oxidative Damage Followed by Inducing Neuronal Differentiation
Source: Front Cell Neurosci. 2019 Sep 3;13:395. doi: 10.3389/fncel.2019.00395 (PMC6733922; doi:10.3389/fncel.2019.00395)
Supplement: Supplementary file 1 [file Data_Sheet_1.pdf]

Berberine protects C17.2 neural stem cells from oxidative damage followed by inducing neuronal differentiation

Jia-Wen Shou [1,2], Chun-Kai Cheung [2], Jian Gao [4], Wei-Wei Shi [4] and Pang-Chui Shaw [1, 2, 3,*]

[1] School of Life Sciences, The Chinese University of Hong Kong, Shatin, Hong Kong, China

[2] Li Dak Sum Yip Yio Chin R & D Centre for Chinese Medicine, The Chinese University of Hong Kong, Shatin, Hong Kong, China

[3] State Key Laboratory of Research of Bioactivities and Clinical Applications of Medicinal Plants (CUHK) and Institute of Chinese Medicine, The Chinese University of Hong Kong, Shatin, Hong Kong, China

[4] Shenzhen Health Development Research Center, Shenzhen, China

[*]Corresponding authors:

Wei-Wei Shi

Shenzhen Health Development Research Center, Shenzhen, China

Tel.: +86-755-83552132; Fax: +86-755-83552132.

Email address: shi1985@outlook.com

Pang-Chui Shaw

School of Life Sciences, The Chinese University of Hong Kong, Shatin, Hong Kong, China,

Tel.: +852 39431363; Fax: +852 26037246.

Email address: pcshaw@cuhk.edu.hk

Supplementary Figure legends

FIGURE 1S| C17.2 cell viability after berberine (0.85, 1.69, 3.38, 6.75, 13.5, 27.0, 54.0 μM) treatment for 12 hours. The data represent the mean ± SEM.

FIGURE 2S| C17.2 cell viability after berberine (0.85, 1.69, 3.38, 6.75, 13.5, 27.0, 54.0 μM) treatment for 24 hours. The data represent the mean ± SEM. *P<0.05, **P<0.01 and ***P<0.001.

Figure 1S

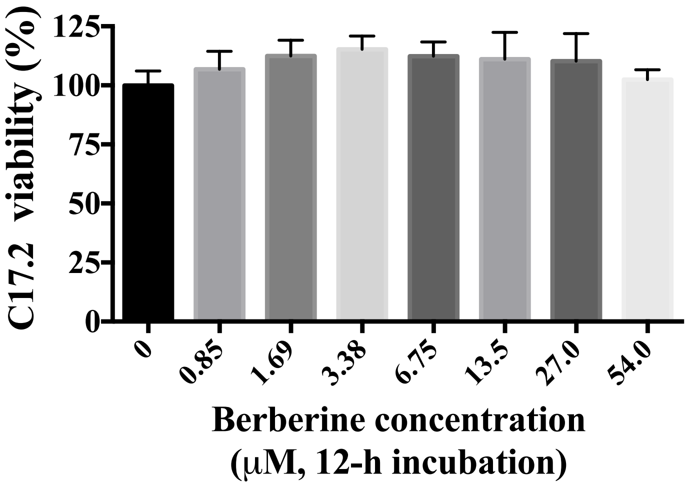
C17.2 viability (%)
125
100
75
50
25
0
0
0.85
1.69
3.38
6.75
13.5
27.0
54.0
Berberine concentration
(μM, 12-h incubation)

Figure 2S

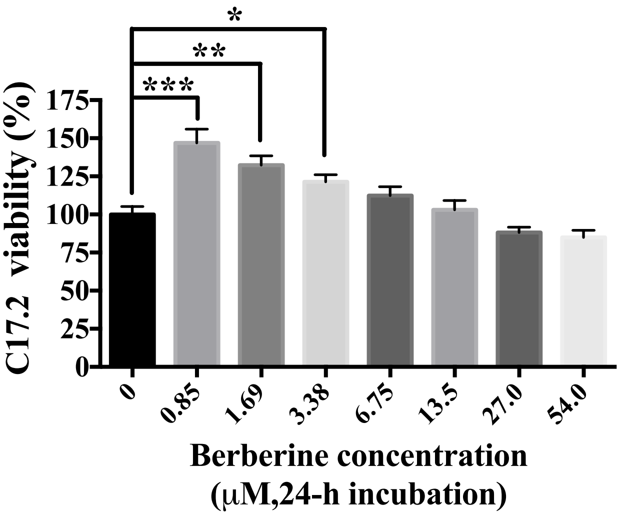
*
**
***
C17.2 viability (%)
175
150
125
100
75
50
25
0
0
0.85
1.69
3.38
6.75
13.5
27.0
54.0
Berberine concentration
(μM,24-h incubation)
